# Supplementary material for: Therapeutic Efficacy of CD34-Derived Allogeneic Dendritic Cells Engineered to Express CD93, CD40L, and CXCL13 in Humanized Mouse Models of Pancreatic Cancer
Source: Vaccines (Basel). 2025 Jul 12;13(7):749. doi: 10.3390/vaccines13070749 (PMC12298679; doi:10.3390/vaccines13070749)
Supplement: Supplementary file 1 [file vaccines-13-00749-s001.zip › vaccines-3675142-supplementary.pdf]

SUPPLEMENTARY TABLES

Table S1: Downregulation of CD34. Flow cytometry results of surface marker expression during CD34+ expansion from D3 to D8 (percentages).

Day	CD34	HLA-DR	CD93	CD45	CD86	CD14	CD40L
**3**	98.9	99.2	0	100	0.2	0.0	0
**8**	28.9	69.9	38.7	100	2.4	1.4	18.0

Table S2: Upregulation of CD14. Flow cytometry results of surface marker expression during monocyte differentiation from D8 to D19 (percentages).

Day	CD34	HLA-DR	CD86	CD14	CD209	CD40	CCR7	CD11c
**19**	2.6	33.0	19.2	22.8	3.2	15.1	12.0	15.6
**22**	0.2	45.4	23.2	24.1	7.1	20.9	13.3	20.9

Table S3. Upregulation of CD11c and HLA-DR. Flow cytometry results of surface marker expression during DC differentiation from D23 to D24 (percentages).

Day	HLA-DR	CD45	CD86	CD14	CD209	CD40	CCR7	CD11c
**23**	49.8	100	50.0	35.1	8.4	57.8	44.3	88.3
**24**	91.8	100	92.2	34.2	4.6	96.5	88.1	95.5

Table S4. TBNK Purity Panel. Flow cytometry results of surface marker expression during DC differentiation from D23 to D24 (percentages).

Day	CD34	CD3	CD16	CD56	CD19	CD8	CD4
**23**	0.4	0	0	0	0	3.0	18.1
**24**	0.6	0	0	0	0	3.0	33.3

Table S5: RT-qPCR Cq Values of Transgenes. Cq values obtained for CD93, CD40L and CXCL13, as well as the reference gene, GAPDH, across different samples. Undetermined Cq values or above 35 values were classified as "Not Detected."

Sample ID	GAPDH Cq	CD93 Cq	CD40L Cq	CXCL13 Cq
**Lentiviral Control DCs**	19.04 ± 0.10	Not Detected	Not Detected	Not Detected
**Engineered DCs**	18.98 ± 0.10	24.02 ± 0.12	25.48 ± 0.10	23.33 ± 0.10

Table S6: Statistical Power Analysis for Figure 6

Experiment / Comparison	Sample Size per Group (n)	Total Events (approx.)	Effect Size (Cohen's d or Hazard Ratio)	Effect Size Description	Estimated Power (%) at α=0.05 (two-sided)	Statistical Test	Notes
Median Survival Time (MST) Control vs Treatment	Control = 7, Treatment = 9	16	0.50 (HR)	50% hazard reduction	~28%	Log-rank test	Small sample size limits power to detect moderate effects
Median Survival Time (MST) Control vs Treatment	Control = 7, Treatment = 9	16	0.40 (HR)	60% hazard reduction	~44%	Log-rank test	Power improves with larger effect size
Median Survival Time (MST) Control vs Treatment	Control = 7, Treatment = 9	16	0.30 (HR)	70% hazard reduction	~66%	Log-rank test	Detecting strong effects possible with moderate power
Figure 6C (Tumor volume reduction)	Control = 7, Treatment = 9	—	2.8 (Cohen's d)	Very large effect size	~82%	Two-sided t-test	Based on prior experience with similar humanized models
Figure 6E (Tumor volume reduction)	Control = 3, Treatment = 3	—	3.1 (Cohen's d)	Very large effect size	~85%	Two-sided t-test	Based on prior experience with similar humanized models

SUPPLEMENTAL FIGURES

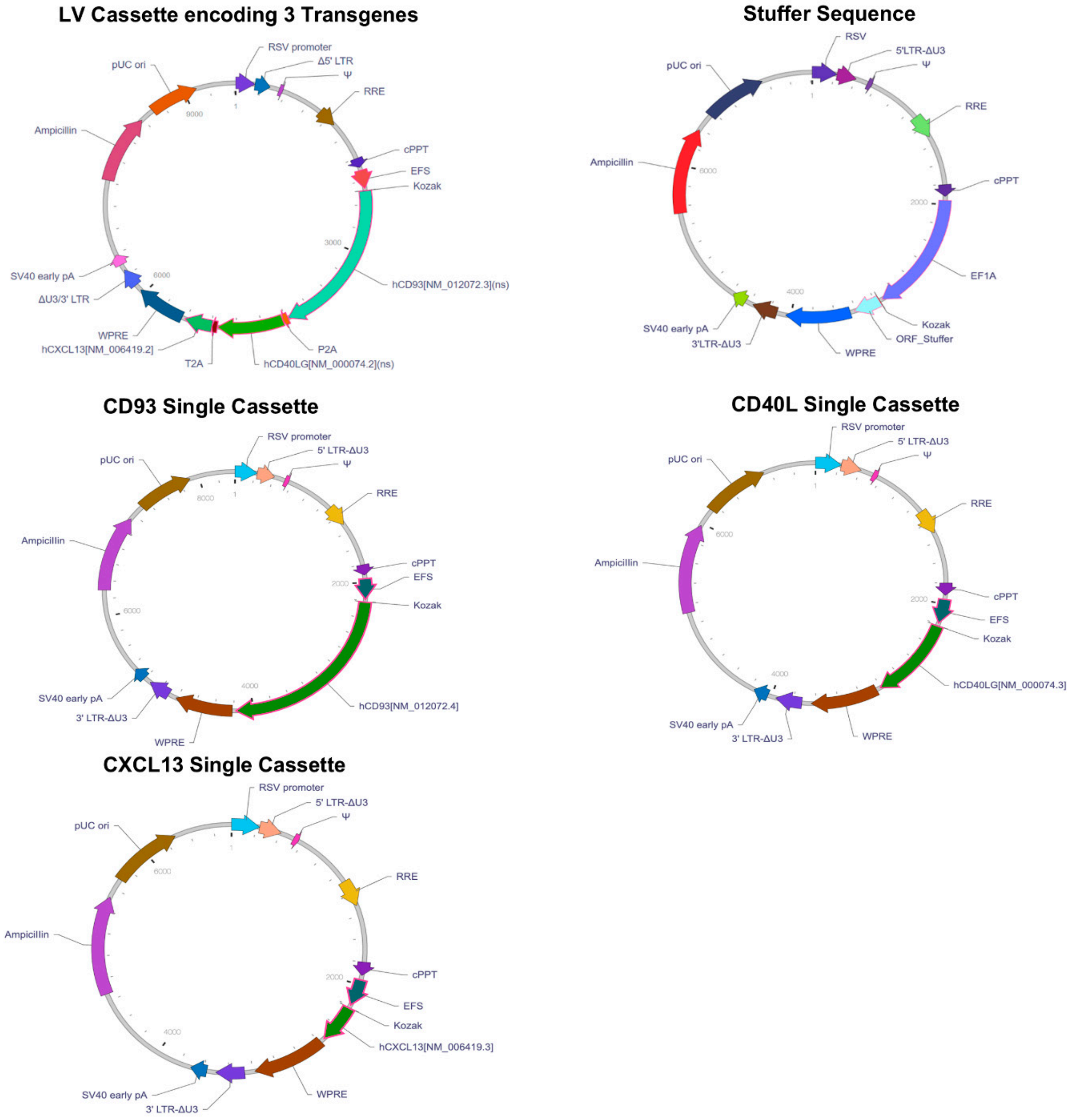

Figure S1. Lentiviral Vector Maps. The LVV open reading frame includes the three genes of interest: CD93, CD40L, and CXCL13, separated by P2A and T2A linkers. Additional features include an EFS promoter and an ampicillin resistance sequence. The stuffer vector contains an EF1A promoter and a stuffer sequence, without any of the three genes present. Additionally, three vectors, each containing a single transgene (CD93, CD40L, or CXCL13), were generated. These three vectors share the same backbone with an EFS promoter and an ampicillin resistance sequence.

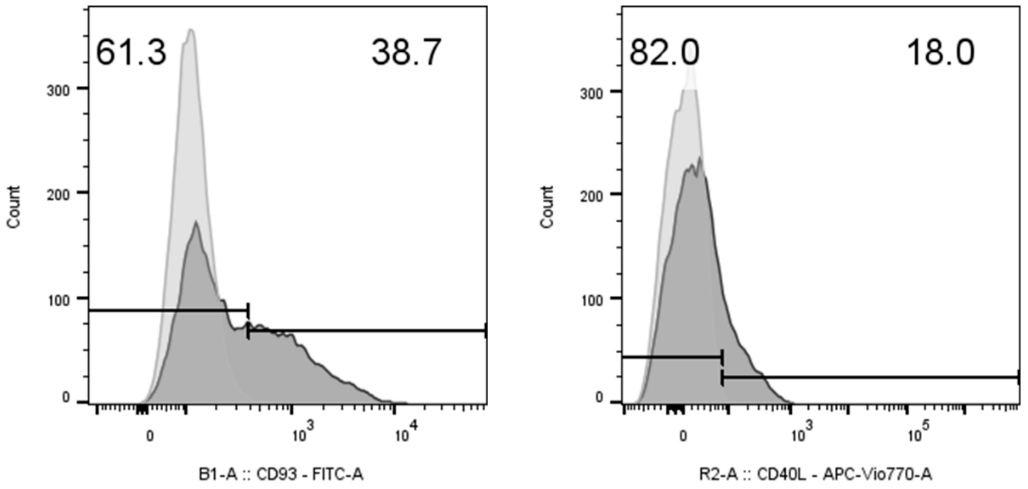

Figure S2. CD93 and CD40L expression in Engineered DCs. Histograms illustrating the CD93 and CD40L expression in engineered DCs following the transduction and differentiation process: 38.7% of cells expressed CD93 while 18% of cells expressed CD40L.
